# Glycolipid composition of the heterocyst envelope of *Anabaena* sp. PCC 7120 is crucial for diazotrophic growth and relies on the UDP‐galactose 4‐epimerase HgdA

**DOI:** 10.1002/mbo3.811

**Published:** 2019-02-25

**Authors:** Dmitry Shvarev, Carolina N. Nishi, Iris Maldener

**Affiliations:** ^1^ Organismic Interactions, Interfaculty Institute of Microbiology and Infection Medicine Eberhard Karls University of Tübingen Tübingen Germany

**Keywords:** *Anabaena*sp. PCC 7120, cyanobacterium, epimerase, glycolipids, heterocyst differentiation, HgdA

## Abstract

The nitrogenase complex in the heterocysts of the filamentous freshwater cyanobacterium *Anabaena*sp. PCC 7120 fixes atmospheric nitrogen to allow diazotrophic growth. The heterocyst cell envelope protects the nitrogenase from oxygen and consists of a polysaccharide and a glycolipid layer that are formed by a complex process involving the recruitment of different proteins. Here, we studied the function of the putative nucleoside‐diphosphate‐sugar epimerase HgdA, which along with HgdB and HgdC is essential for deposition of the glycolipid layer and growth without a combined nitrogen source. Using site‐directed mutagenesis and single homologous recombination approach, we performed a thoroughly functional characterization of HgdA and confirmed that the glycolipid layer of the *hgdA*mutant heterocyst is aberrant as shown by transmission electron microscopy and chemical analysis. The *hgdA* gene was expressed during late stages of the heterocyst differentiation. GFP‐tagged HgdA protein localized inside the heterocysts. The purified HgdA protein had UDP‐galactose 4‐epimerase activity in vitro. This enzyme could be responsible for synthesis of heterocyst‐specific glycolipid precursors, which could be transported over the cell wall by the ABC transporter components HgdB/HgdC.

## 1 INTRODUCTION

*Anabaena* sp. PCC 7120 (also known as *Nostoc* sp. PCC 7120, hereafter *Anabaena* sp.) belongs to a group of multicellular filamentous cyanobacteria that can differentiate and form heterocysts, cells specialized in N_2_ fixation. Upon removal of a source of combined nitrogen, heterocysts arise along the filaments in a semi‐regular pattern, with approximately one heterocyst to ten vegetative cells.

Heterocysts host the extremely oxygen‐sensitive nitrogenase complex (Adams & Duggan, 1999; Kumar, Mella‐Herrera, & Golden, 2010; Maldener, Summers, & Sukenik, 2014; Muro‐Pastor & Hess, 2012; Walsby, 2007). The required microoxic environment in the differentiating cells is achieved by shutting down of oxygenic photosynthesis, activation of respiration, and several morphological changes. The most obvious cellular modification is the synthesis of the heterocyst cell envelope outside of the normal Gram‐negative cell wall (Adams & Duggan, 1999; Kumar et al., 2010; Maldener et al., 2014; Muro‐Pastor & Hess, 2012). This heterocyst envelope consists of two different layers: the outermost exopolysaccharide (hep) layer and the underlying glycolipid (hgl) layer. The hgl layer restricts gas influx into the heterocyst cytoplasm, and the hep layer mechanically supports the hgl layer (Maldener et al., 2014). The glycolipids of the hgl layer (HGLs) are heterocyst specific and can differ in the aglycone length, sugar moiety, or number and type of functional groups (e.g., diol, keto‐ol, and triol) (Bale et al., 2018, 2015; Bauersachs et al., 2009; Gambacorta, Pagnotta, Romano, Sodano, & Trincone, 1998; Gambacorta et al., 1996; Gambacorta, Trincone, Soriente, & Sodano, 1999; Schouten et al., 2013; Soriente et al., 1993).

In *Anabaena* sp., the most abundant HGLs are 1‐(*O*‐α‐d‐glucopyranosyl)‐3,25‐hexacosanediol (HGL_26_ diol) and its 3‐ketotautomer (HGL_26_ keto‐ol) (Gambacorta et al., 1996, 1999). The synthesis of HGLs and deposition of the hgl layer probably constitute a multistep pathway involving products of different genes (Awai, Lechno‐Yossef, & Wolk, 2009; Maldener et al., 2014), and many questions remain open. It is known that a type I secretion system (T1SS)‐like transporter is involved in the efflux of HGLs from the inside of the developing heterocysts to form the hgl layer (Fiedler, Arnold, Hannus, & Maldener, 1998; Maldener, Fiedler, Ernst, Fernández‐Piñas, & Wolk, 1994; Staron, Forchhammer, & Maldener, 2011, 2014). This transporter is composed of the TolC homolog outer membrane protein HgdD, the periplasmic membrane fusion protein DevB, and the inner membrane ABC transporter DevCA. The DevBCA‐HgdD efflux pump is essential for the hgl layer formation and heterocyst function (Fiedler et al., 1998; Staron et al., 2011).

Several homologs of the *devBCA* gene cluster in the genome of *Anabaena* sp. have been identified (Shvarev & Maldener, 2018; Staron, 2012). Some are important for diazotrophic growth and heterocyst maturation (Fan et al., 2005; Shvarev, Nishi, Wörmer, & Maldener, 2018; Staron & Maldener, 2012). The cluster *all5347/all5346/all5345* (*hgdB/hgdC/hgdA*) is of particular interest because the ATPase‐coding gene *devA* is replaced by the *hgdA* gene coding for a putative epimerase. This gene cluster is essential for proper hgl layer deposition and growth of *Anabaena* sp. without combined nitrogen source (Fan et al., 2005; Shvarev et al., 2018), but the functions of the protein HgdA (All5345) was unknown.

Epimerases form a large group of enzymes that can be found in bacteria, animals and plants (Allard, Giraud, & Naismith, 2001). They take part in important metabolic processes, for example, UDP‐galactose 4‐epimerase participates in the Leloir pathway, in which it converts UDP‐galactose to UDP‐glucose (Beerens, Soetaert, & Desmet, 2015; Maxwell, 1957; Wilson & Hogness, 1964). Epimerases mainly constitute dimers, however, other oligomeric states can also be found; the structures of some of these proteins have been resolved (Bauer, Rayment, Frey, & Holden, 1992; Carbone, Schofield, Sang, Sutherland‐Smith, & Ronimus, 2018; Deacon, Ni, Coleman, & Ealick, 2000; Giraud, Leonard, Field, Berlind, & Naismith, 2000). In the present study, we investigated the role of the putative epimerase HgdA in *Anabaena*sp. during diazotrophic growth.

## 2 MATERIALS AND METHODS

### 2.1 Organisms and growth conditions

*Anabaena* sp. PCC 7120 wild‐type and its derivative mutant strains were cultivated in liquid BG11 medium (Rippka, Deruelles, Waterbury, Herdman, & Stanier, 1979) in 100‐ml Erlenmeyer flasks under continuous illumination (17–22 μmol photons m^−2^ s^−1^) at 28°C with shaking at 120 rpm. For RNA isolation, cells were cultivated in 700 ml of nitrate‐free BG11 medium (BG11_0_) supplemented with 2.5 mmol/L NH_4_Cl as a nitrogen source and 5 mmol/L TES buffer (pH 7.8) in 1‐L bottles continuously supplied with CO_2_‐enriched air (2%). Mutant strains were cultivated in BG11 medium supplemented with spectinomycin and streptomycin (2.5 µg/ml each).

For the nitrogen stepdown experiments, cells were washed three times in BG11_0_medium and cultivated afterward in BG11_0_.

All cloning and plasmid maintenance occurred in *Escherichia coli* strains Top10, NEB10, Lemo21 (DE3), and HB101. For triparental mating, *E. coli* strain J53 (bearing the conjugative plasmid RP4), strain HB101 (bearing the helper plasmid pRL528 and the cargo plasmid pRL277 with a fragment of the gene of interest), and wild‐type *Anabaena* sp. were used (Black, Cai, & Wolk, 1993; Elhai & Wolk, 1988; Wolk, Vonshak, Kehoe, & Elhai, 1984) (Tables A1).

The *hgdA* gene for protein synthesis was overexpressed in *E. coli* Lemo21 (DE3) (Table A1).

### 2.2 DNA manipulations

**Figure 1:**
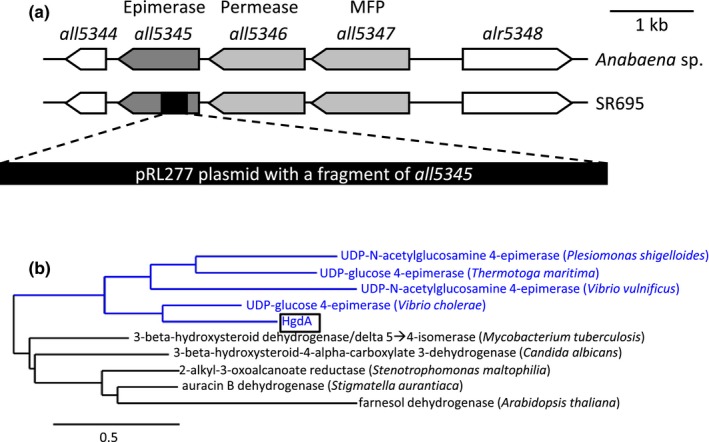
In silico analysis of HgdA. (a) Genomic organization of the *Anabaena*sp. wild‐type and the SR695 mutant strain in the region of the *hgdB/hgdC/hgdA* (*all5347/all5346/all5345*) gene cluster. MFP: membrane fusion protein. (b) Phylogenetic tree of several characterized HgdA homologs found using the PaperBLAST online tool. The maximum‐likelihood tree was constructed with the PhyML program (v3.1/3.0 aLRT) by the web service Phylogeny.fr

To construct an insertion mutant of *hgdA* by homologous recombination, an internal fragment of the gene was amplified by PCR (see Table A2 for primers) with 1 µl of the wild‐type *Anabaena* sp. culture as a template and cloned into the *Xho*I‐restricted suicide vector pRL277 (Table A1) using Gibson assembly (Gibson et al., 2009) (Figure A2a). The resulting plasmid pIM695 was transferred into wild‐type *Anabaena* sp. cells by triparental mating, followed by selection on streptomycin‐ and spectinomycin‐containing BG11 agar plates. In the antibiotic‐resistant *Anabaena* sp. colonies, where a single recombination event between the *hgdA* gene in the genome and its internal fragment in the pIM695 vector had occurred, the *hgdA* gene was disrupted by the pRL277 vector (Figure 1A, A2a). Full segregation of one selected mutant (SR695) colony was confirmed by PCR (Figure A2b) with a small piece of the mutant colony as template.

To localize HgdA in *Anabaena* sp. filaments, a plasmid with a translational fusion of the HgdA C‐terminus with the superfolder GFP (sfGFP) (Pédelacq, Cabantous, Tran, Terwilliger, & Waldo, 2006) was constructed following the method described in (Shvarev et al., 2018). The 3′‐end of *hgdA* and the entire *sfGFP* were amplified by PCR and cloned into the *Xho*I‐restricted suicide vector pRL277 using Gibson assembly. The resulting plasmid pIM717 was transferred into wild‐type *Anabaena* sp. cells using triparental mating, followed by positive colony selection on streptomycin‐ and spectinomycin‐containing BG11 agar plates. *Anabaena* sp. colonies contained the *hgdA*gene fused with *sfGFP* (strain SR717). The fusion was confirmed by PCR.

For complementation of the SR695 mutant, the *hgdA*gene under control of the *glnA* promoter (Valladares, Muro‐Pastor, Herrero, & Flores, 2004) was cloned into the *EcoR*I‐restricted self‐replicating plasmid pIM612, which bears a neomycin‐resistance cassette (Bornikoel, 2018), using Gibson assembly. The resulting plasmid pIM774 was transferred into mutant SR695 cells, and positive colonies were selected on BG11 agar plates containing neomycin, streptomycin, and spectinomycin. The presence of the undisrupted *hgdA* gene in the complemented mutant colonies was confirmed by PCR (Figure A2b).

For overexpression of the *hgdA*gene in *E. coli*, *hgdA*, followed by sequences encoding a Strep‐tag and His‐tag at the 3′‐terminus was cloned into plasmid pET15b (Novagen, Merck) digested with *Nco*I, with help of Gibson assembly to yield plasmid pIM753.

### 2.3 RNA isolation and RT‐PCR

RNA was isolated at different time points after nitrogen stepdown using UPzol reagent (Biotechrabbit, Henningsdorf) according to the manufacturer's instructions from wild‐type *Anabaena* sp. cells grown in bottles as described above. The purity and concentration of the extracted RNA were estimated by electrophoresis and GelQuantNET software (biochemlabsolutions.com). Reverse transcription (RT) reactions were performed using the Applied Biosystems RT‐reaction kit. The primers used for all PCR reactions are listed in Table A2.

### 2.4 Microscopy

For light and fluorescence microscopy, wild‐type and mutant *Anabaena* sp. cells were placed onto agarose‐covered glass slides and observed under a Leica DM 2500 microscope connected to a Leica DFC420C camera or a Leica DM5500 B microscope connected to a Leica monochrome DFC360 FX camera.

Fluorescence of GFP and BODIPY was recorded using a BP470 40‐nm excitation filter and a BP525 50‐nm emission filter. Cyanobacterial autofluorescence was captured using a 50‐nm BP535 excitation filter and a 75‐nm BP610 emission filter. Images were exposed for 80–150 ms in the fluorescence channels. Images of sfGFP and BODIPY fluorescence were taken as Z‐stacks with 0.4‐µm intervals. Z‐stacks were subsequently used to do 3D deconvolution using the integrated function of the Leica ASF software (Leica Microsystems). Images of fluorescence were recolored by the Leica ASF software based on the filters used.

For electron microscopy, cells were fixed and postfixed with glutaraldehyde and potassium permanganate, respectively (Fiedler et al., 1998). Ultrathin sections were stained with uranyl acetate and lead citrate and examined with a Philips Tecnai 10 electron microscope at 80 kHz.

### 2.5 Staining methods for light microscopy

The heterocyst envelope glycolipids were stained with BODIPY (boron dipyrromethene difluoride 493/503, Molecular Probes) that specifically stains neutral lipids (therefore the hgl layer) following the protocol described by Perez, Forchhammer, Salerno, and Maldener (2016).

Briefly, 1 ml of *Anabaena* sp. cell suspension was centrifuged at 4,000 × *g* for 10 min, washed with PBS buffer, and resuspended in 200 µl PBS. BODIPY (1 μl of 50 ng/ml in DMSO) was added. The cell suspension was incubated in the dark for 30 min at room temperature and examined by light and fluorescence microscopy. Fluorescence or phase‐contrast images were captured with a Leica DM 5500B microscope connected to a Leica monochrome DFC360 FX camera.

Cells were stained with alcian blue following the protocol described in (McKinney, 1953). Cell suspensions were mixed with 1.5% Alcian blue in water (at a ratio of 20:1) and incubated at room temperature for 5 min. For triphenyl tetrazolium chloride (TTC) staining, cell suspensions were mixed with TTC solution (0.01% TTC, w/v, in the final mixture) and incubated in the dark for 10 min at room temperature (Fay & Kulasooriya, 1972). Filaments stained with TTC or alcian blue were examined using a Leica DM 2500 microscope connected to a Leica DFC420C camera.

### 2.6 Analysis of heterocyst‐specific glycolipids

Glycolipids were analyzed by thin‐layer chromatography (TLC) as described in (Winkenbach, Wolk, & Jost, 1972) with minor modifications. In brief, wild‐type and mutant cells of equal chlorophyll *a* concentration [measured according to (Mackinney, 1941)] were pelleted and resuspended in methanol–chloroform (1:1) and pelleted again to remove cell debris. The solvents of the supernatant were evaporated under air in a fume hood. Lipids were dissolved in chloroform and applied to a silica‐gel‐coated aluminum plate (Macherey‐Nagel, #818033). Thin‐layer chromatographs were run with a mobile phase composed of chloroform:methanol:acetic acid:water (23:4:2.7:1). Lipids were visualized by spraying the plate with 25%–50% sulfuric acid and exposing it to 180°C for 60–120 s.

### 2.7 Nitrogenase activity

Nitrogenase activity was measured using the acetylene reduction method for cyanobacteria (Bornikoel, Staiger, Madlung, Forchhammer, & Maldener, 2018). Briefly, cultures were incubated in the presence of acetylene for several hours in flasks closed with gas‐tight caps. Anoxic conditions were generated before incubation with acetylene by adding 3‐(3,4‐dichlorophenyl)‐1,1‐dimethylurea (DCMU, 10 µmol/L, in methanol); the sealed flasks were then filled with argon and incubated for 1 hr. For oxic conditions, this step was omitted. After incubation with acetylene, 1 ml of the gaseous phase was taken from each flask, and the amount of ethylene produced was measured by gas chromatography.

### 2.8 Preparation of *Anabaena*sp. vegetative and heterocyst cell lysates

Heterocysts were isolated as previously described (Golden, Robinson, & Haselkorn, 1985; Moslavac et al., 2007). Briefly, after nitrogen stepdown and incubation for 3 days, cells were collected by centrifugation; the pellet was resuspended in 15 ml of ice‐cold 8% sucrose, 5% Triton X‐100, 50 mmol/L EDTA pH 8.0, 50 mmol/L Tris pH 8.0, and 1 mg/ml of lysozyme. The suspensions were mixed vigorously on a vortex shaker for 2–3 min at room temperature. The solution was mildly sonicated with a Branson sonifier (3 × 3 min, 30% duty cycle, 3 output control). Heterocysts were collected by centrifugation at 3,000 × *g* for 5 min at 4°C; the supernatant was the vegetative cell lysate. Heterocysts were washed several times in 8% sucrose, 50 mmol/L EDTA pH 8.0, 50 mmol/L Tris pH 8.0.

To obtain the soluble (cytoplasmic) heterocyst fraction, the heterocyst pellet was resuspended in 5 mmol/L HEPES buffer (pH 8.0) containing 1 mmol/L phenylmethylsulfonyl fluoride (PMSF). The suspension was strongly sonicated (5 × 3 min, 50% duty cycle, 5 output control). The cells were then passed through a French pressure cell (SLM instruments, Inc) at 1,100 Psi 4–5 times. The suspension was centrifuged at 3,000 × *g* for 30 min at 4°C to separate undisrupted heterocysts. Then, the supernatant was centrifuged at 15,000 × *g* for 1 hr at 4°C. The supernatant of this last centrifugation step contained the heterocyst cytoplasmic fraction; the pellet consisted of insoluble debris and membranes.

Samples were analyzed by western blotting with polyclonal antibodies raised against the peptide synthesized from the C‐terminus of HgdA (NH_2_‐CQTKNWLQNTDIQKLVK‐COOH). Peptides were synthesized and antibodies were produced by Pineda Antibody‐service (Berlin). Rabbit polyclonal antibodies raised against the PII protein of *Synechococcus* sp. (Forchhammer & De Marsac, 1994) were used as an internal control. After incubation with antibodies against HgdA, washing in PBS buffer containing 0.05% Tween 20 (Carl Roth) and subsequent incubation with PII antibodies, the membrane was washed again and incubated with secondary peroxidase‐coupled anti‐rabbit IgG antibodies (Sigma A6154). For detection, a Lumi‐Light western blotting substrate (Roche) and a Gel Logic 1500 imager (Kodak) were used.

### 2.9 Overexpression of *hgdA* and purification of HgdA

The *hgdA* gene was overexpressed in *E. coli* Lemo21 (DE3) cells carrying the pIM753 plasmid. Cells were cultivated in 5‐L Erlenmeyer flasks containing 1.5 L LB medium at 37°C with continuous shaking at 120 rpm until they reached an OD_600_ of 0.6. Gene expression was induced by adding isopropyl β‐d‐1‐thiogalactopyranoside (Carl Roth) at a final concentration of 0.1 mmol/L and incubation of the flasks at 25°C overnight with shaking. After induction, cells were pelleted at 7,000 × *g* for 15 min at 4°C, and the pellet was resuspended in lysis buffer (20 mmol/L Tris, 200 mmol/L NaCl, 0.5% Triton X‐100, pH 7.5) containing 1 mmol/L PMSF and 1 mg/ml lysozyme and incubated at room temperature for 1–2 hr. Then, the solutions were sonicated with a Branson sonifier (3 × 3 min, 50% duty cycle, 5 output control) and centrifuged at 17,000 × *g* for 30 min at 4°C. The supernatant, which contained extracted soluble proteins, was used for purification of HgdA by affinity chromatography using a Strep‐column (IBA‐Lifesciences) and Tris buffer (20 mmol/L Tris, 200 mmol/L NaCl, pH 7.5) for equilibration of the column and washing steps; the same buffer containing 2.5 mmol/L desthiobiotin was used for elution. The eluted fractions were pooled and concentrated, and the purity of the HgdA protein was checked by SDS‐PAGE.

HgdA was more highly purified and its oligomeric state was estimated by size‐exclusion chromatography using an ÄKTA chromatography system and a Superdex 75 10/300 column in Tris buffer (see above). To calculate the molecular masses of the proteins in the eluted peaks, a mixture of standard proteins (Gel Filtration LMW Calibration Kit, GE Life Sciences) was run through the column. The fractions corresponding to different peaks of HgdA purification were pooled, concentrated, and analyzed by SDS‐PAGE. The concentration of pure HgdA protein was determined by the Bradford method using Roti‐Quant solution (Carl Roth).

### 2.10 Crosslinking assay

Interacting proteins were crosslinked with suberic acid bis(3‐sulfo‐*N*‐hydroxysuccinimide ester) (BS^3^), which crosslinks epimerases (Timson, 2005). Purified HgdA (5 µmol/L) was incubated with BS^3^ (100 µmol/L) for 30 min at 37°C in 25 µl of Tris buffer (see above). Afterward, the entire sample was used for SDS‐PAGE analysis.

### 2.11 Epimerase activity assay

To test the epimerase activity of HgdA, an established colorimetric glucose oxidase‐horseradish peroxidase (GOD‐POD) coupled assay was used (Beerens, Soetaert, & Desmet, 2013; Moreno, Rodicio, & Herrero, 1981; Pardeshi, Rao, & Balaji, 2017). In brief, 1 mmol/L UDP‐Gal dissolved in 20 mmol/L Tris buffer containing 200 mmol/L NaCl, pH 7.5 was incubated with different amounts of purified HgdA in a total reaction volume of 22 μl at 37°C for 1 or 2 hr. Reactions were stopped and proteins were acid‐hydrolyzed with 3.5 μl of 0.4 N HCl at 100°C for 6 min. The mixture was neutralized with 3.5 μl of 0.4 N NaOH. Aliquots (7.5 μl) were taken from each reaction mixture and applied to a 96‐well plate. The GOD‐POD assay was used to detect released glucose according to the manufacturer's instructions (Sigma‐Aldrich). The reaction was stopped and the color was developed by adding 100 μl of 6 N HCl per well. Afterward, the absorbance at 540 nm was read by a TECAN Spark 10M plate reader.

## 3 RESULTS

### 3.1 The *hgdA* gene product is homologous to NDP‐sugar epimerases

**Figure A1:**
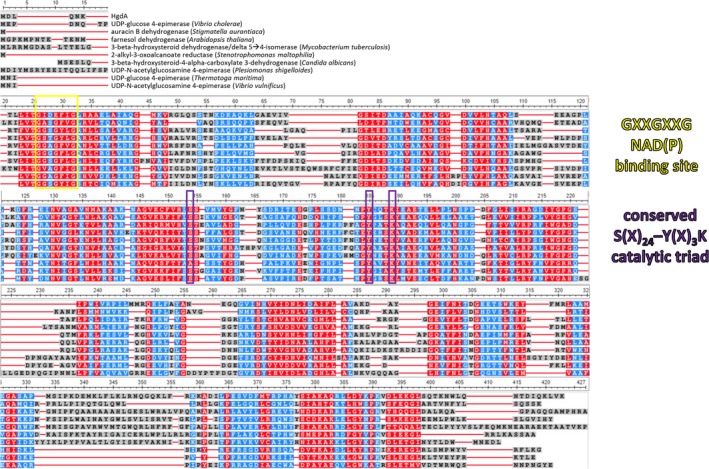
COBALT multiple alignment of several characterized HgdA homologs found using the online tool PaperBLAST. Red, highly conserved residues; blue, less conserved residues; gray, not conserved residues; yellow box, conserved GXXGXXG NAD(P) binding site; violet boxes, residues of the conserved S(X)_24_–Y(X)_3 _K catalytic triad

The *hgdA* gene is the third gene in the previously described cluster involved in heterocyst formation, *all5347/all5346/all5345* (*hgdB/hgdC/hgdA*) [Figure 1a; (Fan et al., 2005; Shvarev et al., 2018)]. It encodes a protein of 333 amino acids with a predicted molecular mass of 36.7 kDa. According to the results of a search using the NCBI BLAST tool, HgdA is a putative nucleoside‐diphosphate‐sugar epimerase belonging to the NAD‐dependent epimerase/dehydratase family of the short‐chain dehydrogenases/reductases (SDR) superfamily. An additional in silico search for homologs of HgdA using the PaperBLAST tool, which searches for homologs of a given protein in published articles (Price & Arkin, 2017), revealed SDRs, including several epimerases, similar to HgdA (Figure A1). However, according to the phylogenetic tree built by the web service Phylogeny.fr (Dereeper et al., 2008; 2010), based on multiple sequence alignments of selected HgdA homologs found by PaperBLAST, HgdA is more closely related to epimerases (Figure 1b).

### 3.2 HgdA protein localizes specifically to heterocysts

**Figure 2:**
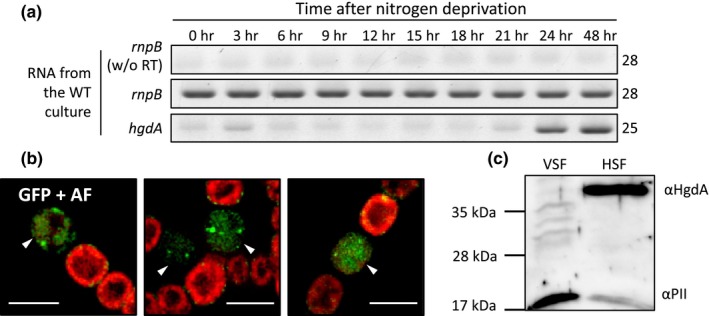
Analysis of *hgdA*expression in *Anabaena*sp. (a) RT‐PCR analysis of time‐dependent *hgdA*expression. *rnpB,* RNase P RNA coding gene, used to ensure that the same amounts of RNA were used for cDNA synthesis in all samples. Numbers at the right indicate the number of PCR cycles. (b) Cellular localization of HgdA in *Anabaena* sp. Fluorescent micrographs of filaments bearing translational fusions of HgdA (All5345) with sfGFP after three days of nitrogen starvation. Green: GFP fluorescence; red: cyanobacterial autofluorescence (AF), white arrowheads, heterocysts. Bar: 5 µm. (c) Western blot analysis of HgdA in vegetative cell fractions (VSF) and heterocyst soluble fractions (HSF). For comparison, antibodies raised against PII were used

To study the function of HgdA, we used semi‐quantitative RT‐PCR to follow the expression of the *hgdA* gene at different time points after transfer of a culture grown on NH_4_^+^ to medium without a combined nitrogen source (nitrogen stepdown) to induce heterocyst differentiation (Figure 2a). The *hgdA* transcript levels were significantly higher at later stages of heterocyst formation, especially at 24–48 hr after nitrogen stepdown. At these time points, the heterocysts were already visible by light microscopy. The upregulation of *hgdA* was notably later than the previously reported upregulation of *devB* (Staron et al., 2011), and the expression pattern of *hgdA*was almost identical to that of *hgdB* (Shvarev et al., 2018).

Since *hgdA* was only expressed under nitrogen starvation, we investigated the localization of the HgdA protein in diazotrophically grown filaments using a fusion protein consisting of HgdA linked at the C‐terminus with sfGFP. The fusion protein localized almost solely to mature heterocysts. GFP fluorescence was equally distributed within the heterocyst and sometimes formed small foci (Figure 2b). This observation is in line with the prediction that HgdA is an epimerase, which is a soluble enzyme.

In western blot analysis, a protein cross‐reacting with the HgdA‐specific antibody was only visible in the sample obtained from the soluble heterocyst fraction, but not in the vegetative cell fraction (Figure 2c) or in membrane fractions (not shown). The PII protein, used as a control, was detected in both samples, but was much more abundant in the vegetative cell fraction (Figure 2c), which is in line with Paz‐Yepez et al. (Paz‐Yepes, Flores, & Herrero, 2009), who demonstrated downregulation of the PII‐encoding gene *glnB*in *Anabaena*sp. heterocysts.

### 3.3 The *hgdA*gene is essential for diazotrophic growth

**Figure 3:**
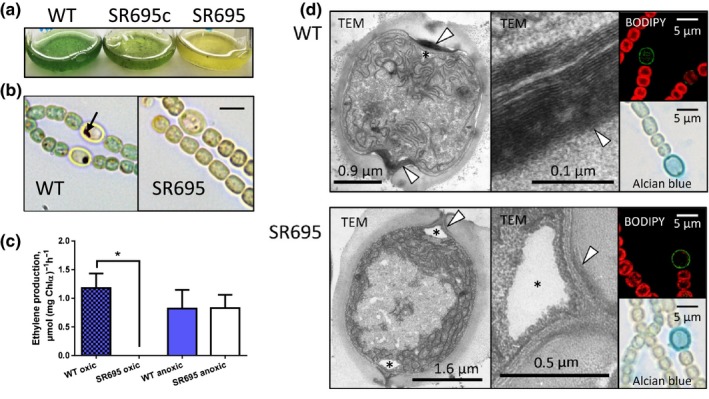
Phenotypic analysis of mutant SR695*.*(a) Growth of wild‐type (WT), mutant SR695, and the mutant complemented with the *hgdA* gene (SR695c) in liquid medium for 3 days without combined nitrogen source. (b) Triphenyl tetrazolium chloride **(**TTC) staining of filaments of wild‐type and mutant SR695. Arrows: dark formazan crystals of reduced TTC under microoxic and reducing conditions necessary for nitrogenase complex activity. Bars: 4.5 µm. (c) Nitrogenase activity in wild‐type and mutant SR695 cells under oxic and anoxic conditions assayed by acetylene reduction. Shown are mean values ± standard deviation of two experimental replicates. *p < 0.05, Student's *t* test. (d) Heterocyst cell envelope. Left panels: transmission electron micrographs (TEM) of wild‐type and mutant SR695 heterocysts; white arrowhead: hgl layer; star: cyanophycin granule; right panels: micrographs of wild‐type and mutant SR695 stained with BODIPY or alcian blue

**Figure A2:**
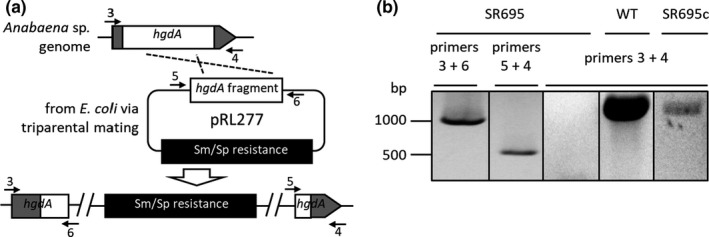
Construction and segregation of the *hgdA*mutant. (a) Construction of an *hgdA* mutant via homologous recombination. Arrows with numbers, primers used for the genotypic analysis of the mutants (see also \ Table A2). (b) Segregation of the *hgdA*mutant analyzed by PCR. SR695: mutant SR695; wt: wild‐type; SR695c: SR695 mutant complemented with the *hgdA* gene. Primer numbers correspond to those depicted in A

To investigate the function of the *hgdA* gene in more detail, we created a mutant of this gene in *Anabaena* sp. by inserting an antibiotic resistance gene via homologous recombination (Figure 1a, A2a). The mutant, SR695, was completely segregated (Figure A2b). In medium with a combined nitrogen source, mutant SR695 did not differ from the wild‐type in cell and filament morphology or in growth. However, the mutant was not able to grow diazotrophically, even though the filaments formed heterocysts after nitrogen stepdown (Figure 3a,b,d). The mutant SR695 was complemented by introducing the self‐replicating vector pIM612 (Bornikoel, 2018) carrying the full‐length *hgdA* sequence under control of the P*_glnA_* promoter (Valladares et al., 2004). The complemented mutant (SR695c) clearly grew better than the mutant SR695 at 7 days after nitrogen stepdown (Figure 3a).

### 3.4 The aberrant cell envelope of heterocysts of mutant SR695 cannot provide microoxic conditions for nitrogenase activity

We investigated whether heterocysts of the mutant SR695 provide microoxic conditions necessary for nitrogenase activity. We incubated mutant and wild‐type cultures with triphenyl tetrazolium chloride **(**TTC) (Fay & Kulasooriya, 1972) and observed the dark crystals of reduced TTC only in wild‐type heterocysts (Figure 3b). Lack of dark TTC crystals in mutant heterocysts indicated that their inner environment was oxic.

Mutants with defects in heterocyst envelope layers only have nitrogenase activity when incubated under anoxic conditions (Ernst et al., 1992). We assayed nitrogenase activity under oxic and anoxic conditions based on the measurement of acetylene reduction (Bornikoel et al., 2018). The mutant SR695 had nitrogenase activity only under anoxic conditions, whereas the wild‐type had nitrogenase activity under both oxic and anoxic conditions (Figure 3c). Hence, the mutant heterocysts did not provide the microoxic conditions required for nitrogenase activity.

We analyzed the heterocyst envelope in more detail. We were able to detect both cell layers (hep layer stained by alcian blue and hgl layer stained by BODIPY) of the mutant heterocysts with standard labeling methods and light microscopy (McKinney, 1953; Perez et al., 2016), and the heterocysts appeared normal at this resolution (Figure 3d). However, wild‐type and mutant heterocysts differed in ultrathin sections analyzed by transmission electron microscopy (TEM). The mutant heterocysts lacked the typical laminated hgl layer, and at the heterocyst–vegetative cell connections at the polar neck regions it was thinner compared to the wild‐type (Figure 3d). These observations are comparable with those of the *hgdA*mutant FQ1647 (Fan et al., 2005). We did not find any structural differences in the hep layer or in the ultrastructure of vegetative cells between wild‐type and mutant SR695.

**Figure 4:**
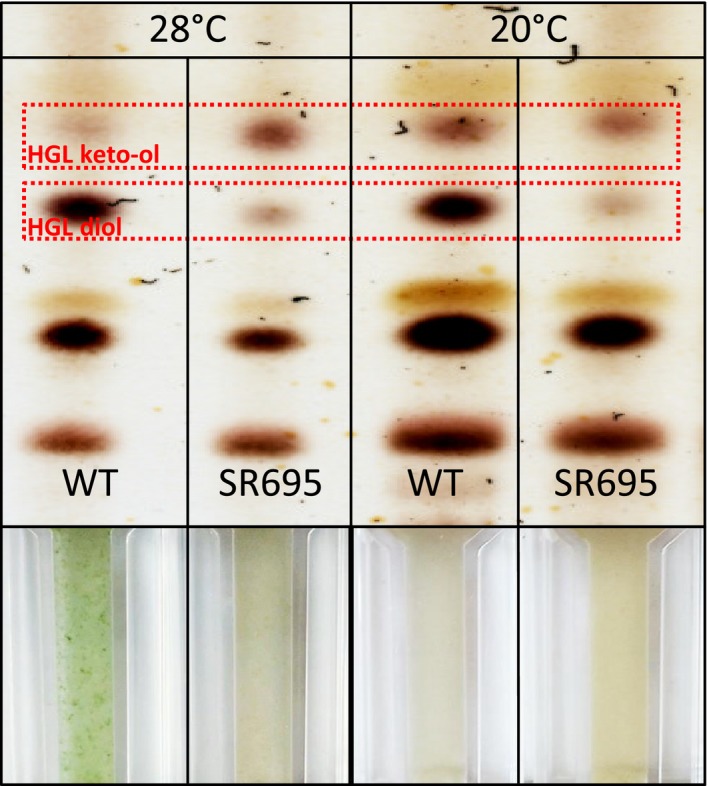
Comparison of the glycolipid composition of wild‐type and mutant SR695 heterocysts. TLC analysis of lipids obtained from whole cell extracts of cultures incubated at the indicated temperatures. Red boxes: heterocyst‐specific glycolipids; lower panels: photographs of the respective cultures

We analyzed the glycolipid composition of mutant and wild‐type hgl layers using TLC of methanol extracts of both strains after nitrogen stepdown. We analyzed the content at two temperatures (20 and 28°C) because the ratio of the major HGL forms can vary at different temperatures (Bauersachs, Stal, Grego, & Schwark, 2014; Wörmer, Cires, Velazquez, Quesada, & Hinrichs, 2012). Both wild‐type and mutant extracts contained the major HGLs, HGL_26_ keto‐ol and HGL_26_ diol (Perez, Wörmer, Sass, & Maldener, 2018) at both temperatures (Figure 4). However, the diol:keto‐ol ratio of wild‐type and mutant SR695 differed. At 28°C, the diol:keto‐ol ratio of wild‐type heterocysts was higher than that of the mutant (Figure 4). At 20°C, the wild‐type contained more of the keto‐ol form than at 28°C. Nevertheless, the wild‐type was still different from the mutant, which did not show a temperature dependent ratio change (Figure 4). As previously reported, a mutant in the upstream gene *hgdB* shows a similar phenotype at 28°C (Shvarev et al., 2018). We also found that at 20°C, differences in growth between wild‐type and mutant SR695 were not as prominent as at 28°C (Figure 4, lower panels).

### 3.5 The protein HgdA is soluble and forms dimers in vitro

**Figure 5:**
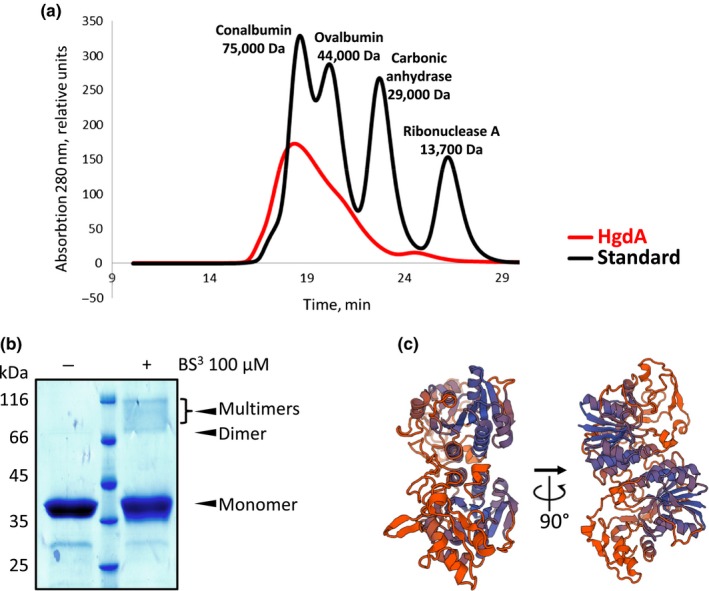
Analysis of the oligomeric state of HgdA. (a) Size‐exclusion chromatography elution profile of recombinant HgdA and a mixture of standard proteins. Peak fractions containing HgdA corresponds to the dimer. (b) SDS‐PAGE of recombinant HgdA cross‐linked with BS^3^. (c) Predicted structure of the HgdA dimer based on the structure of the homologous enzyme WbgU (a UDP‐GalNAc 4‐epimerase), created using a Swiss model online tool

For the biochemical characterization of the HgdA protein, we overexpressed the gene in *E. coli* and purified the protein by affinity chromatography, followed by size‐exclusion chromatography. The major peak of HgdA in the size‐exclusion chromatography elution profile corresponded to the dimeric form; additional peak shoulders, probably representing monomeric and other oligomeric forms of HgdA, were also present (Figure 5a).

On SDS‐polyacrylamide gels, the band of purified HgdA consisted of the monomeric form. However, when purified HgdA was incubated with the amino‐reactive cross‐linker BS^3^, which forms covalent bonds between interacting proteins, also dimeric and other oligomeric forms were detected (Figure 5b).

We modeled the structure of HgdA using the Swiss model online tool (Waterhouse et al., 2018) based on its closest homolog with a solved structure, namely the UDP‐GalNAc 4‐epimerase WbgU. The modeling revealed that HgdA probably forms dimers (Figure 5c), in agreement with the results of size‐exclusion chromatography and crosslinking experiments.

### 3.6 HgdA fulfills a UDP‐galactose 4‐epimerase function in vitro

**Figure A3:**
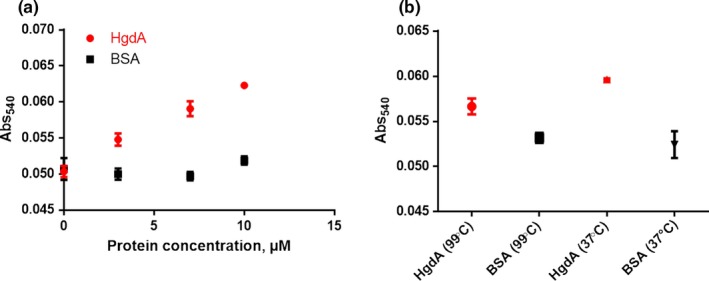
Enzymatic activity of HgdA. (a and b) Glucose oxidase‐horseradish peroxidase assay of UDP‐galactose 4‐epimerase activity of recombinant dimeric HgdA. HgdA (red) at the indicated concentrations was incubated at 37°C (a) or at concentration 10 µmol/L at 37°C or 99°C (b) with 1 mmol/L UDP‐galactose, and UDP‐glucose production was measured (expressed in units of absorbance at 540 nm, Abs_540_). Each point represents the mean value ± standard deviation of two experimental replicates. Bovine serum albumin (BSA) was used as a negative control (black). In (a), a representative of two independent experiments is shown

Based on the sequence similarity of HgdA to UDP‐galactose 4‐epimerase, we tested whether HgdA converts UDP‐galactose to UDP‐glucose (Moreno et al., 1981). The enzyme catalyzed the conversion at a rate of approximately 30–40 nmol min^−1^ nmol HgdA^−1^ depending on the protein concentration. UDP‐glucose production by HgdA increased when higher concentrations of the enzyme were used; the activity was considerably lower when tested at 99°C (Table 1, Figure A3).

**Table 1:** Enzymatic activity of HgdA

	Relative activity, AU	Standard deviation, AU	# replicates
HgdA, reaction at 37°C	1.196	0.061	6
BSA, reaction at 37°C	1.026	0.028	6
HgdA, reaction at 99°C	1.104	0.023	2
BSA, reaction at 99°C	1.035	0.014	2

Note

Glucose oxidase‐horseradish peroxidase assay of UDP‐galactose 4‐epimerase activity of recombinant HgdA. HgdA at the concentration of 10 µmol/L was incubated with 1 mmol/L UDP‐galactose at indicated temperatures for 2 hr, and UDP‐glucose production was measured. Bovine serum albumin (BSA) was used as negative control. Shown is the relative enzymatic activity in arbitrary units (AU) with the standard deviation of indicated experimental replicates.

UDP‐galactose 4‐epimerases use NAD as a cofactor, which is constantly bound in the conserved cofactor‐binding glycine‐rich site in the Rossmann fold (Allard et al., 2001; Beerens et al., 2015; Bellamacina, 1996; Rossmann, Moras, & Olsen, 1974). However, we were unable to extract or detect NAD from the enzyme using standard protocols (Creuzenet, Belanger, Wakarchuk, & Lam, 2000). In place of the conserved NAD‐binding motif GXXGXXG, the HgdA sequence has a GIDEFIG motif, with the second glycine replaced by glutamate (Figure A1). An NCBI BLAST search showed that such a motif is also found in HgdA homologs in several other cyanobacteria.

## 4 DISCUSSION

One of the main events in heterocyst maturation is the formation of the heterocyst‐specific envelope. A variety of enzymes participate in this process, including those that are responsible for the synthesis and transport of the envelope components (Fan et al., 2005; Fiedler et al., 1998; Huang et al., 2005; Maldener, Hannus, & Kammerer, 2003; Nicolaisen, Hahn, & Schleiff, 2009; Shvarev et al., 2018; Staron et al., 2011). In this study, we investigated the function of the putative epimerase HgdA (Figure 1) in heterocyst formation. Our results partially confirmed previous findings (Fan et al., 2005; Shvarev et al., 2018), and in addition described the enzymatic activity of HgdA.

Transcripts of *hgdA* were found only when the heterocysts were almost completely mature (24–48 hr after nitrogen stepdown; Figure 2a). These time points were markedly later than activation of the *devB* gene, which encodes the membrane fusion component of the efflux pump transporting HGLs (Fiedler et al., 1998; Staron et al., 2011). However, the expression patterns of the *hgdB*and *hgdC* genes (Shvarev et al., 2018) are similar to that of *hgdA*, which indicates that products of the *hgdBCA* gene cluster are formed at the same time even if they probably do not comprise an operon since they were complemented separately (Fan et al., 2005) and that the proteins might work together. The localization of HgdA in the cytoplasm of mature heterocysts (Figure 2b, c) confirms its specific importance for these differentiated cells and demonstrates that HgdA is a soluble protein, as expected from the in silico analysis of its sequence.

Our mutant SR695, like the transposon‐insertion mutant of this gene (Fan et al., 2005) showed a Fox^−^ phenotype, that is, the inability to grow diazotrophically under oxic conditions. Since nitrogenase activity was detectable under anoxic conditions, this mutant shows a phenotype, which is specific for mutants with an impaired heterocyst envelope (Figure 3a–c).

Although the hgl layer was present in the mutant SR695 (Figure 3d), its defect allowed oxygen to enter the heterocyst. The main difference between the mutant and wild‐type was in the HGL composition (Figure 4). Specifically, the aberrant ratio of the two major HGLs in the mutant, with an excess of HGL_26_ keto‐ol, seemed to be critical for hgl layer formation and heterocyst function at 28°C. The same aberrant HGL ratio, which causes a Fox^−^ phenotype, in an *hgdB*mutant has been found (Shvarev et al., 2018); this finding along with the results of our expression studies might indicate that products of the *hgdB*,* hgdC*, and *hgdA*genes work cooperatively. Most UDP‐galactose 4‐epimerases form dimers or other oligomers (Allard et al., 2001), but they can also function in a monomeric state (Nayar & Bhattacharyya, 1997). Our size‐exclusion chromatography, crosslinking, and modeling results indicated that the main active states of HgdA are probably dimers (Figure 5).

Purified HgdA has typical UDP‐galactose 4‐epimerase activity in vitro (Table 1). Compared to other known UDP‐galactose 4‐epimerase activities, this activity was in the lower range, with some epimerases having activities several times less and others hundreds of times more than that of HgdA (Agarwal, Gopal, Upadhyaya, & Dixit, 2007; Chung, Ryu, & Lee, 2012; Guevara, El‐Kereamy, Yaish, Mei‐Bi, & Rothstein, 2014; Pardeshi et al., 2017; Shin et al., 2015). Since we were unable to detect or extract NAD from the purified active HgdA protein, we assume that the altered NAD‐binding sequence, with glutamate replacing glycine, captures more tightly NAD.

The function of an epimerase in HGL synthesis has not been described so far. But based on our results, we suggest that HgdA converts UDP‐galactose, which could derive from thylakoid degradation in heterocysts, to UDP‐glucose. By a still unknown mechanism, the different activated sugar epimers determine the ratio between HGL diol and HGL keto‐ol.

Nevertheless, other HGL diol biosynthetic pathways independent of HgdA must be present because mutant SR695 heterocysts contain a small amount of the HGL diol (Figure 4), which is not sufficient to support heterocyst function. The similarity of the phenotypes of the *hgdA* mutant and *hgdB* mutant (Shvarev et al., 2018) suggests that HgdA and HgdBC closely cooperate, but further investigation is required.

Altered HGL diol:keto‐ol ratios have been described in other situations. For instance, during growth at higher temperatures, cyanobacteria produce higher amounts of HGL diols (Bauersachs et al., 2014; Wörmer et al., 2012), which might protect heterocysts from gas penetration under these conditions. When HGL keto‐ols are prevalent and the amount of HGL diols is lower, the heterocyst cell envelope might lose its gas tightness at higher temperatures; at lower temperatures, when amounts of the keto‐ol form increase, the envelope retains its gas tightness.

**Figure 6:**
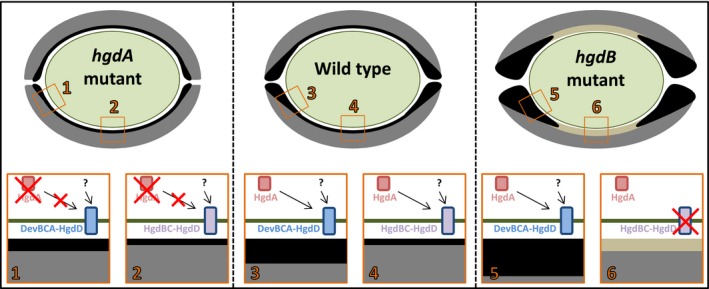
Comparison of hgl layers of heterocysts from wild‐type, *hgdA* mutant, and *hgdB* mutant of *Anabaena* sp*.*The wild‐type heterocyst possesses normal hgl (black) and hep (gray) layers. The *hgdA*mutant heterocyst has a much thinner hgl layer. The *hgdB*mutant heterocyst (Fan et al., 2005; Shvarev et al., 2018) has a thicker hgl layer at the polar regions but it is replaced by an amorphous unstructured layer at the lateral sides of the cell. Green line, cellular membranes and cell wall. Lower panels show the putative interplay between the different means of HGL synthesis and transport involving HgdA and transporters DevBCA‐HgdD (Staron et al., 2011) or HgdBC‐HgdD (Shvarev et al., 2018). Arrows from HgdA show the route of HGLs, produced with help of HgdA, to a transporter (DevBCA‐HgdD or HgdBC‐HgdD). Arrows from the question marks indicate routes of HGLs produced independently of HgdA (probably at earlier stages of heterocyst development)

The deposition of the HGLs in the wild‐type and in the *hgdA* or *hgdB*mutant (Fan et al., 2005; Shvarev et al., 2018) differs (Figure 6). The wild‐type forms a normal hgl layer around the entire heterocyst using two exporter systems, namely DevBCA‐HgdD (Staron et al., 2011) mostly at the polar neck regions and HgdBC‐HgdD (Shvarev et al., 2018) at the lateral sides. In the *hgdB*mutant, the hgl layer is replaced by an amorphous layer at the lateral sides because the HgdBC transporter is lacking, and the hgl layer is thicker at the polar regions because of excess substrate for DevBCA‐HgdD (HGLs that are not transported by HgdBC‐HgdD but are still synthesized). In the *hgdA*mutant, both transporters are present, but because HGL production is deficient, the hgl layer is much thinner than in the wild‐type.

In conclusion, our results indicate that the epimerase HgdA takes part in the synthesis of the HGL diol form, thereby controlling the HGL keto‐ol:diol ratio, and probably works at the late stages of heterocyst development and fine‐tunes the proportion of HGL in the heterocyst envelope. At this stage, much UDP‐galactose (HgdA substrate) must be available in the heterocysts; this substrate originates from the main components of thylakoid membranes, digalactosyldiacylglycerol (DGDG) and monogalactosyldiacylglycerol (MGDG) (Boudière et al., 2014; Maida & Awai, 2016; Yuzawa et al., 2014), which are degraded during heterocyst maturation. HgdA probably takes part in the synthesis of HGLs at some point, and a transporter composed of HgdBC and HgdD (TolC homolog) might export HgdA products directly or sequentially to the heterocyst cell envelope.

## CONFLICT OF INTERESTS

The authors declare no conflict of interest.

## AUTHORS CONTRIBUTION

DS and IM designed the experiments; DS and CNN performed experiments; DS and IM analyzed the data and wrote the manuscript; all authors read the final manuscript; IM supervised the project.

## ETHICS STATEMENT

None required.

## Data Availability

The data will be available on request from the corresponding author.

## APPENDIX 1

**Table A1:** *Anabaena* sp. PCC 7120, *Escherichia coli* strains, and plasmids used in the work

Strain or plasmid	Reference or source
*Anabaena* sp. PCC 7120 wild‐type	Rippka, Deruelles, Waterbury, Herdman, and Stanier (1979)
SR659	This work
SR717	This work
NEB10	New England Biolabs
Top10	Invitrogen
HB101 (pRL528)	Wolk et al. (1984)
J53 (RP4)	Wolk et al. (1984)
Lemo BL21 (λDE3)	Novagen/Merck
pIM659	This work
pIM717	This work
pRL277	Black, Cai, and Wolk (1993)
pET15b	Novagen/Merck
pIM753	This work

**Table A2:** Oligonucleotides used in the work. All primers were purchased from Sigma‐Aldrich

Number	Primer	Purpose
1	5′TGATAATAAGCGGATGAATGGCAGAAATTCGATATCTAGATCTTGCGTGCAGCTGAGTTG3′	Cloning of *hgdA* fragment for mutant generation
2	5′CGCCGGCATGTCCCCCTGGCGGACGGGAAGTATCCAGCTCGACGGGTGGAGAATTTAAGG3′	Cloning of *hgdA* fragment for mutant generation
3	5′TCTCCTGGGTTGAGAATG3′	*hgdA* forward (fw) primer
4	5′ATGCAGTCAGAGAAATGAG3′	*hgdA* reverse (rv) primer
5	5′TTAAACGCCTGGTGCTACGC3′	pRL277 fw primer
6	5′GGCCTCTTCATCGGGAATGC3′	pRL277 rw primer
7	5′CAGCTGAGTTGGCGATAG3′	*hgdA* RT‐PCR fw primer
8	5′GGACAATTGCGCTGTATG3′	*hgdA* RT‐PCR rv primer
9	5′ATAATAAGCGGATGAATGGCAGAAATTCGATATCTAGATCGATCGCATCACCGAATCC3′	Cloning of *hgdA‐sfGFP* fusion
10	5′CTGTAAATAATTCTTCACCTTTTGAAGAGCCTCCTCCACCTTTAACTAGCTTTTGAATATCGGTATTTTG3′	Cloning of *hgdA‐sfGFP* fusion
11	5′CAAAATACCGATATTCAAAAGCTAGTTAAAGGTGGAGGAGGCTCTTCAAAAGGTGAAGAATTATTTACAG3′	Cloning of *hgdA‐sfGFP* fusion
12	5′CATCGCCGGCATGTCCCCCTGGCGGACGGGAAGTATCCAGCTCGATTATTTATATAATTCATCCATACCATGTGTAATA3′	Cloning of *hgdA‐sfGFP* fusion
13	5′CTAGACGGATATCCCGCAAGAGGCCCTTTCGTCTTCAAGGGATTTTATGTCAAAGTTGACCCCTATG3′	Cloning of P_glnA_‐*hgdA* construct for complementation of the *hgdA* mutant
14	5′CATCAATTCCAGTAATCAGCAGAGTTTTATTTTGCAAATCCATTGTTACTCCTTCTCTGCCAATTTC3′	Cloning of P_glnA_‐*hgdA* construct for complementation of the *hgdA* mutant
15	5′GAATTTAAGAAATTGGCAGAGAAGGAGTAACAATGGATTTGCAAAATAAAACTCTGCTGATTACTG3′	Cloning of P_glnA_‐*hgdA* construct for complementation of the *hgdA* mutant
16	5′CATTAAAGCTTATCGATGATAAGCTGTCAAACATGAGAATTCTATTTAACTAGCTTTTGAATATCGGTATTTTG3′	Cloning of P_glnA_‐*hgdA* construct for complementation of the *hgdA* mutant
17	5′CCTCTAGAAATAATTTTGTTTAACTTTAAGAAGGAGATATACCATGGATTTGCAAAATAAAACTCTGCTGATTACTG3′	Cloning for synthesis of the HgdA protein
18	5′TGCTGTGATGATGATGATGATGGCTGCTGCCTTATTTTTCGAACTGCGGGTGGCTCCAAGCGCTGTGGTGGTGGTGGTGGTGTTTA3′	Cloning for synthesis of the HgdA protein
